# Meeting the Needs of Rural Veterans: A Qualitative Evaluation of Whole Health Coaches’ Expanded Services and Support during COVID-19

**DOI:** 10.3390/ijerph192013447

**Published:** 2022-10-18

**Authors:** J. Hale-Gallardo, Consuelo M. Kreider, Gail Castañeda, Kelsea LeBeau, Deepthi S. Varma, Cheri Knecht, Diane Cowper Ripley, Huanguang Jia

**Affiliations:** 1 Veterans Rural Health Resource Center-Salt Lake City (VRHRC-SLC), Office of Rural Health, Veterans Health Administration, Salt Lake City, UT 84148, USA; 2 Veterans Rural Health Resource Center-Gainesville (VRHRC-GNV), Office of Rural Health, Veterans Health Administration, Gainesville, FL 32601, USA or; 3 Department of Occupational Therapy, University of Florida, Gainesville, FL 32611, USA; 4 Department of Epidemiology, University of Florida, Gainesville, FL 32611, USA; 5 Department of Biostatistics, University of Florida, Gainesville, FL 32611, USA

**Keywords:** healthcare, social determinants of health, military, telehealth, allied health, pandemic

## Abstract

The purpose of this qualitative study was to explore perspectives of Whole Health (WH) coaches at the Veterans Health Administration (VHA) on meeting the needs of rural Veterans during the COVID-19 pandemic. The evaluation design employed a qualitative description approach, employing focus groups and in-depth interviews with a convenience sample of WH coaches across the VHA system. Fourteen coaches who work with rural Veterans participated in either one of three focus groups, individual interviews, or both. The focus group data and in-depth interviews were analyzed separately using thematic analysis, and findings were then merged to compare themes across both datasets. Four primary themes were identified: bridging social risk factors for rural Veterans, leveraging technology to stay connected with Veterans at-a-distance, redirecting Veterans to alternate modes of self-care, and maintaining flexibility in coaching role during COVID-19. One overarching theme was also identified following a post-hoc analysis driven by interdisciplinary team discussion: increased concerns for Veteran mental health during COVID-19. Coaches reported using a variety of strategies to respond to the wide-ranging needs of rural Veterans during the pandemic. Implications of findings for future research and practice are discussed.

## 1. Introduction

Health coaches represent one of the most rapidly growing allied health professions in the United States [1,2,3,4,5]. Health coaches partner with clients to identify wellness goals and bolster the knowledge and skills needed to manage chronic conditions [2,6,7,8,9,10,11]. They achieve this by facilitating “a respectful, peer-like conversation that evokes versus admonishes, refrains from giving advice or lecturing, and leads with empathy, while building rapport and tracking progress” [12] (p. 5). Health coaches add value to a healthcare model that increasingly recognizes patients as “the most underutilized resource” [13] (p. 27) and shared decision-making as an essential foundation of patient-centered healthcare [14,15,16].

In 2018, the United States (US) Department of Veterans Affairs (VA) Veterans Health Administration (VHA) Office of Patient Centered Care and Cultural Transformation began implementing the Whole Health (WH) initiative, an integrative health approach empowering and equipping Veterans to take charge of their health and wellbeing [17,18,19,20]. WH coaches have been at the front lines of efforts to transform the VHA from a disease-focused healthcare model to one of “creating health” in partnership with patients [8,21,22]; as such, they are helping to shift the conversation from “What is the matter with you?” to “What matters to you?” [23,24]. As of 2021, the VHA had trained close to 2000 Whole Health coaches [25], and by mid-2022, the Whole Health Coaching course had been provided to more than 3215 VHA staff members (personal communication). 

Many WH coaches work with rural Veterans, an often hard-to-reach population sharing substantial barriers and constraints to healthcare access while representing a wide range of service eras and sociodemographic diversity [26,27,28,29,30,31,32,33,34]. While rural Veterans tend to be at higher risk for preventable diseases, they also experience differences in healthcare access compared with urban or suburban-dwelling Veterans, resulting in disparities in disease burden [29,32]. Health coaching can be especially beneficial for Veterans who live in rural areas where comprehensive services are often limited and who may bear additional comorbidities [9,10,11,27,34].

The coronavirus (COVID-19) pandemic disrupted the traditional face-to-face delivery of VHA health and wellness programs [35,36]. While there is evidence demonstrating that Veterans in the VHA’s WH program experienced wide ranging biosocial impacts during the pandemic [8], there is a need for more understanding about how coaches attempted to meet the needs of Veterans residing in rural areas under conditions of COVID-19. The purpose of this study was to explore perspectives of Whole Health (WH) coaches at the Veterans Health Administration (VHA) on meeting the needs of rural Veterans during this time of national crisis.

## 2. Materials and Methods

This paper is based on work conducted as part of an evaluation design to examine coaches’ efforts to extend WH to rural Veterans throughout the VHA. This project was sponsored by the VHA Office of Rural Health. The evaluation was deemed a quality improvement project by VA guidance [36] and was considered exempt from review as per North Florida/South Georgia Veterans Health System Research Service and the University of Florida Institutional Review Board guidelines.

### 2.1. Project Design and Population

WH coaches are employees of the VHA, and a subset of these employees are Veterans. Qualifications for WH coaches typically include a bachelor’s or higher degree in a behavioral or social science, and specialized experience in helping clients reach wellness goals utilizing motivational interviewing and shared decision-making health coaching techniques [37]. In addition, a growing number of health coaches obtain a National Board Certification of a Health and Wellness Coach (NBC-HWC) [38].

Whole Health is a VA wide initiative; there are no particular inclusions or exclusions for Veterans participating in WH coaching aside from being enrolled for healthcare at the VHA. Some common health conditions of WH Veteran users may include chronic musculoskeletal pain and psychiatric conditions such as depressive disorder, anxiety disorders, and PTSD [39,40,41].

This evaluation design is based in a qualitative description approach [42], commonly used in healthcare research where the goal is to discover the ‘who’, ‘what’, and ‘where’ of experiences, and gain insights from informants. This qualitative approach employed focus groups and in-depth interviews. Focus groups (FGs) were conducted to generate discussion among WH coaches surrounding barriers and facilitators of reaching rural Veterans during the pandemic. Follow-up individual interviews were utilized to further probe how WH coaches were engaging rural Veterans during the pandemic. Our primary objective of this evaluation was to understand the ways in which coaches addressed the needs of rural Veterans during the pandemic, focusing on the level of qualitative description [43]. Altogether, we conducted three FGs with a total of 11 participants (FG1 = 4, FG2 = 4, and FG3 = 3 participants, respectively) and nine follow-up interviews, of which eight had participated in FGs. A total of 14 WH coaches informed this analysis.

During the planned analysis, data indicating the reasons why the coaches addressed Veterans’ needs during the pandemic began to emerge from the data. Therefore, a post-hoc analysis was conducted to identify patterns specific to the reasons why coaches engaged in actions that were identified in the primary analysis.

### 2.2. Data Collection

#### 2.2.1. Focus Groups

The evaluation team developed the FG guide in collaboration with VHA WH stakeholders. Prior to initiating FGs, 33 potential participants who had expressed interest in the quality improvement project during a WH national Community-of-Practice Coaching call were sent a brief, web-based email questionnaire to elicit participant characteristics, coaching experience, and the percentage of rural Veterans coached.

Three FGs were conducted in August and September 2020 during national lockdowns due to the pandemic. All FGs were conducted virtually using Zoom videoconferencing technology. Two to three project team members were present during each FG to facilitate discussion and help with troubleshooting issues. Each FG lasted 60 min. All FGs were audio recorded and participants provided verbal consent specifying their participation was voluntary and confidential.

#### 2.2.2. In-Depth Interviews

The semi-structured interview guide was informed by findings from the FGs and explored in greater depth (1) the role of coaches during the pandemic; and (2) barriers or facilitators coaches experienced when working with rural Veterans.

FG participants, as well as those who had previously expressed interest, received an invitation for a follow-up one-on-one interview. Interviews took place between December 2020 and January 2021; this was a period when VHA facilities were beginning to re-open critical, in-person services to Veterans while also continuing to maintain options for socially distanced healthcare through telehealth modalities.

All interviews were via Zoom calls. As with the FGs, multiple team members were present during each interview to facilitate discussion and troubleshoot any technical issues. Interview participants gave verbal consent to participate and to be audio and video recorded for the purpose of supporting analysis. Each interview lasted approximately 60 min.

### 2.3. Analysis

#### 2.3.1. Demographic Questionnaire

Participant demographic characteristics, coaching experience, and percentage of time coaching rural Veterans were summarized utilizing descriptive statistical analysis.

#### 2.3.2. Focus Groups

Transcripts from FGs were generated from audio recordings using the VA’s centralized transcription service program. A thematic analysis was performed on the transcribed data to identify latent patterns in the data and develop preliminary themes, following established guidelines for thematic analysis [44]. Three coders with qualitative research training independently coded the FG data and then compared codes to achieve consensus for final themes and subthemes that represented meaning within the data set in relation to the question about WH coaches’ experience reaching and engaging rural-dwelling Veterans during COVID-19.

#### 2.3.3. In-Depth Interviews

Interview transcripts were generated from the audio recordings. Thematic analysis of the in-depth interviews was informed by the coding framework developed in the analysis of the FGs. This resulted in a new coding framework agreed upon by three members of the project and was inputted into an excel matrix. The final coding framework resulted in a validation of a priori themes of interest, refined themes initially identified from the FGs, and new themes that emerged after researchers read all transcripts. Rigor was enhanced by presentation of preliminary findings to the VHA WH coaching leads.

After completing coding for the FG and interview data, the evaluation team worked together to review and refine results from both datasets. After several iterations, the final output of our analyses were themes, or interpretive concepts, around experiences of WH coaches reaching and engaging rural Veterans during the COVID-19 pandemic. Multiple rounds of comparison, reorganization, and refinement occurred before finalizing our findings.

#### 2.3.4. Post-Hoc Analysis

Through team discussion, primary themes were discussed and constantly compared with the data. This resulted in one overarching theme, whereby representative quotes were threaded throughout the primary findings.

## 3. Results

Participating coaches (14) hailed from nine states within the US representing the Northeast, Southeast, Midwest, and West. Participants were primarily female (66.7%). Coaches’ Veteran status was primarily non-Veteran (64%). Participants who reported practicing WH coaching for more than one year (67%) had an average of 2.1 years of coaching experience. All participants reported their coaching was conducted via telehealth since COVID-19, with more than half the participants reporting ≥50% of their caseload comprised of rural Veterans.

FGs and follow-up interviews generated rich discussions about participants’ experiences engaging rural Veterans with the WH program and coaching during the pandemic. Four primary themes were identified: bridging social risk factors for rural Veterans, leveraging technology to stay connected with Veterans at-a-distance, redirecting Veterans to alternate modes of self-care, and maintaining flexibility in coaching role during COVID-19. The overarching theme was: increased concern for Veteran mental health during COVID-19.

### 3.1. Bridging Social Risk Factors for Rural Veterans

A major theme that emerged from the data was the urgency to address social risk factors and bridge gaps in support of rural Veterans during COVID-19. Subthemes included: lack of access to healthcare resources; lack of access to basic needs (e.g., food and transportation); increased risks for housing displacement; and increased risks for social isolation.

Participants described heightened concern for helping rural Veterans access fundamental resources within their local communities during the national US emergency response in March of 2020. In the words of one participant:

*I was scrambling, home-based primary care was scrambling, we were all scrambling to find a list of resources to get to these Veterans, because it was like, there’s no clinic. Like there’s nowhere for them to get, you know, there was no CVS [i.e., pharmacy, drugstore]. There’s no place to get the flu shot. There’s no place to get [undecipherable], there’s, like, all these things…, and then, on top of it all, we have COVID-19.*

(Interview ID#8, Female Coach, West US.)

Coaches we interviewed felt that while rural Veterans had the advantage of being almost “naturally socially distanced” due to the nature of being in a rural area, it was the challenges of meeting basic needs that caused coaches the most concern during the initial period of the pandemic.

For some rural Veterans, standing financial constraints were compounded by decreased access to food and transportation. One participant expressed how these standing constraints intersected in the lives of some rural Veterans “*[when] they’re already hurting for cash… [and they need to] make an extra trip to go get healthy food and drink, it’s… [the ability] to be able to [have the transportation to] go [is the barrier].*” (FG1, Female Coach, Midwest US.)

Another participant described how her clients were trying to ascertain how they would access basic foodstuffs in locations where store shelves were insufficiently stocked because of the severe wildfires that hit the Western US in 2020 in the midst of the pandemic:

*The problem with not having access to healthy foods, especially during the pandemic, [is that] the shelves are empty. I had two patients… in [area] which is mountains… [and] when it was the fires and everything, their grocery stores were empty… so those are the things that, that our rural [Veterans]… are dealing with. Because, between the fires and the pandemic, they’re not getting the shipments… I know that one lady, the one Veteran that I was dealing with last week… she was able to get a ride from a nephew, because she doesn’t drive, to go a little bit further and find a farmers market; so she was able to get some things; but the grocery store, it didn’t have anything [in stock].*

(Interview ID#8, Female Coach, West US.)

Moreover, this coach highlighted how important it was for her to stay connected to her rural Veteran clients during a time when many people in her region had been displaced by natural disaster. She described not wanting to “lose track” of rural Veterans who were at risk of housing displacement, and leveraging community connections to identify and relay information about specific resources needed by the Veterans:

*I’m like, we don’t want to lose track of our [rural Veterans]. Because if they’re displaced… it wasn’t like we were going to find them easily… And it was like trying to figure out, okay, what resources do we have for me to give them [her rural Veteran clients]? So, it was another thing where it was important to be connected to the community… I was getting the information, to be like, ‘okay… for people who have horses’… there was like whole teams of people who were taking their trailers to go save people’s horses. It is important to be… making those community connections, at any chance, any turn that you can, because in times of emergency, you might be able to find out a little bit more.*

(Interview ID#8, Female Coach, West US.)

Coaches also reported that Veterans they worked with had to travel further to acquire food and were compelled to rely on alternative forms of transportation to travel there, in a context where previous transport options were non-existent or unavailable. Consequently, the local suspension of transportation services also translated into reduced healthcare access for this population:

*[For my] rural patients, I do have several patients who have been impacted a lot by COVID, just simply transportation. You know, when public transportation stops, at a certain point, and then they depend on other kinds of transportation, those are patients who are having trouble just if they have to come to a regular appointment. Like, to get labs drawn. They literally can’t, because the rural transportation has stopped, and they don’t drive.*

(FG2, Female Coach, Southeast US.)

Coaches working with rural-residing Veterans during this time described how important it was to maintain connections with them to try to address the gaps they were facing in meeting basic needs. 

In addition to the challenges around access to healthcare, transportation, and food, as well as increased risks for housing instability, coaches reported that COVID-19 also increased existing rural Veteran risks for social isolation. COVID-19 impacted the ability to meet in-person, something participating coaches noted rural-residing Veterans had expressed enthusiasm for:

*I work at 2 different CBOCs [i.e., community clinics]… At the CBOC which was completely rural…they were glad to have us there. They were glad to have something offered, so they were much more inclined to want to do something [with the WH program]… We went to a coffee talk a few months ago [pre-Covid19]… [and] from my CBOC, it was 35 miles away. Of course, we had Veterans coming in from way past that. And the question was asked: ‘What would it be like for you if we had a Whole Health van that came out to you, at least once a month’, and you saw 150 hands raise up and go—‘Yeah, we would love that’.*

(FG2, Male Coach, Veteran, Midwest US.)

Consequently, through social distancing protocols and suspension of WH group coaching opportunities, Veterans faced increased social isolation. One coach described the impact of the closure of spaces on her rural Veterans who had become accustomed to gathering for group coaching sessions:

*We have a couple ranches here, and so a lot of the times, when I would do my group sessions, I would take them to the ranch… they could also, on the side, do some equine therapy or other things that the ranch offers. [The ranch] cater[s] directly to our Veteran community. Every Wednesday is for women only, so we do a lot of things just for the women on Wednesdays… [and the ranch has] dinners on Thursdays, and Veterans are allowed out there at any time.… [But] they won’t let me have the groups at the ranch now…so that’s very hard, because these people really were getting a lot out of being together.*

(FG1, Female Coach, Midwest US.)

### 3.2. Leveraging Technology to Stay Connected At-a-Distance

Another important theme that emerged was staying connected with rural Veterans at-a-distance by leveraging technology. Staying connected became a priority for coaches working with rural Veterans in the context of lockdowns and social distancing. Coaches described rapidly pivoting to alternative modalities at the VHA to try to ensure uninterrupted access to WH, initially via telephone and then later through the VHA’s video telehealth platform, Veterans Video Connect (VVC):

*Within a week’s time we had flipped all of our face-to-face coaching, and individuals that were signed up for [in-person] Intro to Whole Health were switched over to telephone,…’VVC’ was going to be a part of how our VA did outreach, but it had not been implemented yet, so we… had to quickly implement… [using telephone to reach Veterans].*

(FG3, Female Coach, West US.)

While video telehealth had been part of the VHA for several decades now, WH had relied more on face-to-face contact with Veterans. However, coaches reported that “*COVID really just jumped ‘VVC’ into overdrive*” (FG1, Female Coach, West US.). They also noted that not all Veterans were benefitting equally from the video telehealth platform, especially rural Veterans:

*… As we’re all experiencing right now, we’re not really meeting the needs of [all] our Veterans, because [some] don’t have internet access [and] without having the internet, [rural Veterans] are not able to participate [in Whole Health].*

(FG1, Female Coach, West US.)

Another participating coach explained why some rural Veterans were not able to benefit from the virtual platform for delivering WH, ranging from the lack of broadband connections in rural areas and a lack of appropriate devices, to an insufficient amount of technology literacy:

*There were quite a few Veterans that just didn’t have internet. They didn’t have computers, laptop, or a PC… all they had was their phone… They were either… more frontier [with less reliable internet connection]… [or] older and being a little bit more rural, [they] preferred telephone. They didn’t want to mess with the VVC. They didn’t like it… the bandwidth didn’t work… or they couldn’t figure out their camera… [or the] microphone. Or they just flat out didn’t have a computer and they were trying, you know, to have a doctor’s appointment with their phone.*

(Interview ID#14, Female Coach, West US.)

For those rural Veterans without reliable internet, coaches reported that their options “dwindled”:

*It’s the ones that don’t have internet and are really, really, rural, that I struggle with the most as a health coach—and feeling, ‘how much can I… help them, when they want to work on these things [that are offered virtually]?’… my resource list… dwindles down a lot.*

(FG1, Female Coach, Midwest US.)

To help bridge the gap for these rural Veterans, coaches described the ways in which VHA was increasing its capacity to bridge the digital divide:

*… the VA has just done a big switch here within the last several months, where all video appointment consults… go… to a ‘digital divide consult’… the social worker connects with the Veteran [and asks:] “Do you have a personal device, your cell phone or do you have a computer, or do you not have any [device]?” to try to explore that. And [they ask about] comfort levels if they do have a cell phone or a laptop. If they… have… Wi-Fi or internet, then we can go ahead and get them set up to get their test call and try it out and see if they like it. The majority of them don’t [have a device], so… [we put in a request] to get them a VA-issued iPad… And this was a topic point before COVID19, [but] really it has [been] accentuated during COVID19.*

(FG3, Female Coach, West US.)

Once connected to the internet, coaches also described interdisciplinary efforts to ensure that WH could reach Veterans; for example, in the case of a rural Veteran with traumatic brain injury and a visual and an auditory disability, who needed special technologies to hear and communicate, the coach worked with the Veteran’s audiologist and an interdisciplinary team of providers to troubleshoot how to provide WH to him, “*even though he can’t leave the home and he can’t hear or see*”. (Interview ID#15, Male Coach, Veteran, Southeast US.)

Coaches reported that in some cases, rural Veterans who had internet access but were averse to learning the new technology, or preferred something they considered more convenient, opted for continuing their coaching via the telephone. One coach described a Veteran who lived and worked in a rural area, who would “*schedule his coaching sessions via telephone on his lunch hour… [from an] oil field, so he would literally be… an hour from any… town or any type of… access to anything*”. (Interview ID#14, Female Coach, West US.)

While for some rural Veterans, the lack of broadband connection and/or aversion to using video telehealth modalities made connecting with Veterans via VVC challenging, others felt that the video technologies were expanding the potential for a kind of ‘face time’ that did not rely on being in person. As a coach explained, “*So now that everybody is on VVC… the persons I coached said—”Can we do VVC instead of telephone, so I can see you? I said—Okay.” So we switched that to VVC right now.*” (FG2, Female Coach, Midwest US.)

### 3.3. Redirecting Veterans to Alternate Modes of Self-Care

With COVID-19 limiting gatherings, coaches reported working with rural-dwelling Veterans to find a way to shelter-in-place and practice self-care, despite the isolation. One participant described helping a Veteran problem-solve to find meaningful activities that could be conducted within her own home and with her grandchildren:

*[As a nearby gym was closing]… Veteran said, ‘I could go to Fort [name]’, which is two hours away. [But then Veteran says], ‘I can’t do that’. So, she’s been doing a lot of the home exercising on her own… she’s been doing a lot of self-care. [She said] “You know, I can spend my time out in the garden, keep myself busy, have the grandkids come over and they’ll help out… help me build a tomato [garden].” She’s been a lot more [focused on] self-care [at home than before pre-COVID].*

(Interview ID#1, Male Coach, Veteran, Midwest US.)

In a similar vein, another coach also spoke of doing more coaching and troubleshooting with rural Veterans, supporting their wellness, physically and mentally:

*During COVID I [have done] a lot of coaching, even more than usual, related to ‘how do you keep even doing anything at all?’ Physically, mentally, trying to stay well, when you have a hard time getting into a facility…and that facility meaning a gym or a workout center, or a clinic, but especially health-related things. And lots of really, really small towns or Veterans that are maybe 100 miles away from the closest decent-sized town.*

(FG1, Female Coach, Midwest US.)

Participants also described innovating on WH content that would address the self-care needs of rural Veterans for whom access barriers were exacerbated and developed creative strategies for engaging Veterans in WH. One coach marveled at her success in adapting what was formerly an online yoga session to a phone call:

*A lot of them are suffering from low back pain… [I ask] “Well, have you ever thought about or would you ever be interested in learning [yoga]?” So I actually end up coaching yoga over my phone, and they do it. And… it’s amazing to me, without seeing yoga, just talking yoga, how much they can learn to do, or they’ll say “yes, well, I have my hands here, and I’m standing up” or “I’m folding forward at the waist”… it’s pretty interesting!*

(FG1, Female Coach, Midwest US.)

Sharing calendar schedules filled with new course offerings that ranged from online mindfulness groups to exercise groups to remote cooking classes, another coaching participant described the virtual wellness classes that he offered weekly in addition to coaching:

*I think we’ve done a great job as I offer three mindfulness classes a week and on Tuesdays I have an all-female class… we’ve been offering live [online] yoga classes, live Tai Chi classes, plus ones that are pre-taped. So I think we’ve done a phenomenal job really reaching out to those that may not be working that just want something to do… If they have a low back pain, [I suggest] let’s try the yoga or Tai Chi… [offered] four or five times a week through VVC. So I think we’ve done a phenomenal job really reaching out to those Vets… [we have] all the way up from beginners the to the advanced groups… it gives them [something] to keep… active.*

(Interview ID#13, Male Coach, Veteran, Southeast US.)

Coaches described how the virtual platform allowed for unprecedented participation for rural Veterans in wellness programs:

*N* *ow with COVID, [we] turned just about everything we have on [to] VVC… we find that there is more rural Veteran involvement… those Veterans, I find, have very big commitments [to an online exercise program], so they are very committed, and a lot of them go through, you know, health coaching as well.*

Coaches also described reaching out to rural-residing Veterans with a variety of other resources, such as an app in support of self-care and mental health during COVID-19:

*I don’t want to overwhelm them, but I tell them that I’m going to send them some interesting things. Like the apps. We got a COVID coach app that’s awesome. I love that app. And so, I’ll send them some information, and you know, the people that really, really want to make a change in their life are excited about all that.*

(Interview ID#11, Female Coach, Midwest US.)

### 3.4. Maintaining Flexibility in Coaching Role during COVID-19

Coaches described reaching out to rural Veterans during a time of heightened isolation, but they also reported that not all Veterans were immediately receptive. For some Veterans, the pandemic itself was a rationale for keeping their distance and not engaging in WH programming:

*I’ve had several Veterans who just have kind of said ‘now is not the time for this. I just need to get [through this], you know I can’t be bothered’… That’s always disappointing but understandable. I mean, the truth is we do what we need to do to get through [things] in difficult times. And so you always leave the door open… I usually ask permission and say, “Can I call you in 30 days just to check in and see how you’re doing?” No one’s ever said no to that. So that’s nice.*

(Interview ID#2, Female Coach, Southeast US.)

Considering the heightened social isolation, participants discussed how they adjusted their role as a coach during the pandemic to meet rural Veterans’ most pressing needs:

*One of the things we had started when COVID first hit,… we started outreaching as much as we could to every Veteran that we serve. We just started doing cold calls. Part of it was to check in to say, “Hey, we’re here, we’re alive, we haven’t forgotten you.” And the other is to see if there’s anything they’re interested in,… to see what they needed from us, whether it’s clinical, whether it’s ‘I just need an ear to talk to, because I’m lonely, stuck out here in the middle of nowhere’.*

(FG2, Male Coach, Veteran, Midwest US.)

This same coach reported that his ability to establish rapport with rural Veterans was enhanced by the same isolation his Veteran clients were facing:

*In some ways, [the isolation] makes it easier once you make the connection. When you make the connection, now they have somebody to talk to. They will admit that at times it can get very lonely out there, and they just want to talk to somebody, but they don’t trust enough people to talk to them. So, when they can make that connection, even if it’s over a phone—honestly, most of my rural coaching is done over the phone—they just want to be heard.*

(Interview ID#1, Male Coach, Veteran, Midwest US.)

During the pandemic, some coaches described being one of the main points of contact and one of the only “*regular appointments [rural Veterans] are getting, because they can’t get here [to the facility]*”. (FG2, Female Coach, Southeast US.)

This was especially true during moments of crisis when many people were unsure about their risks for exposure. One coach described using her coaching role as a way to provide social and medical support for rural Veterans:

*They don’t want to come out at all. So… like you know, they’ve been… complaining [about symptoms], and I’m like, “You need to come to the emergency room, or you need to come in.” So I’m calling the RN: “Can you call them?… And it comes from that relationship, the importance of building relationships, especially with rural [Veterans] is important. For every single Veteran, but I feel like especially for rural Veterans, you have to have that relationship.*

(Interview ID#8, Female Coach, West US.)

This coach also explained that she found her coaching role to be an opportunity to help alleviate heightened anxieties during the pandemic:

*Every once in a while, if they’re coming in [to the facility] to take a COVID test, those are the things… they need support in… You know, thinking you have COVID is scary.*

(Interview ID#8, Female Coach, West US.)

Coaches also described being nimble in modifying their typical coaching protocol as the stressors of life under pandemic conditions increased for Veterans. For example, one coach expressed adapting his coaching protocol to for a Veteran “in need of someone to talk to”:

*[I coached a rural-dwelling Veteran] who was a single father and struggling… he was not working and [was] living off about 1400 dollars a month with a 14-year-old daughter that he was not having a good relationship [with]… Even though he wanted a weight loss goal… [as] we started developing some action plans…it seemed like every week, the primary conversation was his challenges with finances and being a provider for his daughter, he was so thankful. He said, “You calling me, I appreciate that. I needed someone to talk to because I think I’m pulling my hair out. She won’t wash the dishes. She won’t listen to me. She won’t do anything…” So he just needed someone to talk to… it really got to where it wasn’t coaching anymore. It was just being someone to actually listen to him. And because of his frustrations, I don’t mind being that that person, at points. It is outside the coaching role… but as a Veteran, I have a passion for other Veterans.*

(Interview ID#15, Male Coach, Veteran, Southeast US.)

This Veteran coach further described how maintaining dialogue to stay connected to Veterans outside of the coaching relationship was important during the pandemic for some Veterans experiencing substance use issues:

*We had to discontinue the coaching session… they need the services that we can’t offer [under coaching]… [the] prime goal is to manage their sobriety… So I have two of them that… I’m still talking to, but [have not] actually coached them at this point; they’re actually back into the inpatient services. I had a Veteran who had a mental health breakdown and so his Whole Health goal was to connect back with his primary care for his mental health condition before continuing coaching. So, we are their connection, [the connection] is still there.*

(Interview ID#15, Male Coach, Veteran, Southeast US.)

In addition, this coach reported staying abreast of Veterans who were also patients of the VHA’s Mental Health service. He followed their charts for evidence of missed appointments and used wellness checks as a touchstone for them:

*… If I’m working with [rural Veterans] that have a high-risk flag [on their medical record], and I haven’t heard from them two weeks… I’m going to do a wellness check. I’m going to call them and ask them, you know, “Are they okay? [I’ll] leave a couple of voicemails, do a chart review, and see if their mental health providers are… being able to contact them.” But it’s things like that, just making sure that those Veterans that I am charged for working with [are OK].*

(Interview ID#15, Male Coach, Veteran, Southeast US.)

Overall, participating coaches highlighted heightened flexibility in their roles as an aid for maintaining relationships with rural Veterans during the pandemic.

## 4. Discussion

This evaluation examined the experiences of WH coaches engaging rural Veterans during the COVID-19 pandemic. From their perspectives, key social determinants of health (SDOH) [45] were negatively impacted for many rural dwelling Veterans during the pandemic in the areas of social and community context, health and health care, and neighborhood and built environment. Coaches demonstrated heightened resourcefulness and ingenuity in attempting to bridge the social risk factors that were exacerbated for rural dwelling Veterans by the pandemic. We found that WH coaches expanded their services and support to provide emotional, informational, and instrumental support [46,47,48] to ameliorate the challenging social conditions faced by their rural Veteran clients. Moreover, WH coaches actively sought to address SDOH by helping to troubleshoot rural Veterans’ most pressing needs, which included healthcare access and transportation, food availability, communication barriers, recreational opportunities, and social support.

Participating WH coaches also extended their services and supports in response to what they perceived as social isolation and its negative impacts on the Veterans they served. Our findings are consistent with those reported in a recent study investigating COVID-19 impacts on Veterans’ health and wellbeing [8]. These researchers identified negative emotional and psychological impacts of COVID-19 to include Veterans’ feelings of social isolation and stress brought about by constraints to daily routines, as well as lack of in-person social contact with others, reduced transportation access, food insecurity, and job loss and financial stresses [8].

We also found that coaches attempted to support Veterans in navigating SDOH-related challenges, while remaining cognizant of the limits of their scope of practice as health coaches. Our findings echo the work of Jordan (2021) who asserted that, during times of crisis, it is appropriate for coaches to adopt a broader public health lens [12]. Coaches, in their training for evoking their clients’ self-reflection toward challenges experienced, can readily expand their coaching dialogue to support clients’ SDOH-related challenges [12], such as those reported by our participating coaches. As such, expanding coaches’ skills for supporting Veterans’ social and public health needs is warranted. Coaches can support these needs through facilitated, evocative, and supportive discussions that increase the Veteran’s self-awareness regarding SDOHs that impact health; coaches can also employ their strengths-based approach to further bolster existing competencies and support skill development [12] in skills that are needed to bridge gaps in key social areas that impact health.

While the behavioral, social, economic, environmental, and occupational factors that impact health have typically fallen outside the domain of the conventional biomedical system [49], unlike most healthcare systems, the VHA is under a statutory commitment to address both medical and non-medical needs of Veterans. Given that life-course disparities tend to accumulate in rural settings [50], a multimodal (mind, body, and spirit) patient-centered approach, as is facilitated by WH coaches, may prove especially valuable for improving the quality of life for rural Veterans, especially in the context of factors that influence health and wellbeing beyond the clinical setting [51].

While working within the VA’s interdisciplinary team-based model of care, coaches in this evaluation drew upon their networks to enact what has been called a ‘science of cooperation’ that partners across health, social services, and local communities [52]. Coaches had to consider more than individual risk factors when seeking to understand health promotion—indeed, a whole-community approach was needed. As such, coaches modified their strategies in support of rural Veterans, adopting a wider perspective of practice to bridge the gaps for rural Veterans that had been exacerbated by the pandemic.

This wider perspective of practice also included innovating with the use of technologies to extend their reach to rural Veterans. Prior to COVID-19, the primary strategies used by coaches to introduce Veterans to WH relied on face-to-face, in-person interactions. Although the VA has long been at the vanguard of developing and implementing tele-based delivery of healthcare services to Veterans [53,54,55,56,57,58,59,60,61], WH coaches had not formerly relied on telehealth approaches and had to pivot quickly to incorporate various technologies and resources into their daily work with Veterans, representing a substantial shift for the WH initiative.

The transition of health coaching to a tele-approach raises salient social science questions when we consider health coaches’ work through the lens of affective labor [62]. Affective labor describes forms of caring labor, including in healthcare services, that relies on human contact, caring and interaction, where the results or outcomes are intangible products, such as feelings of ease, well-being, and satisfaction [62]. WH coaches reported that rural Veterans found themselves experiencing insufficient contact with friends, family members, neighbors, and society at large, exacerbating stressors, and they worked to mitigate these stressors through forms of caring labor. While the COVID-19 lockdown precluded in-person coaching interactions, our findings indicate that WH coaches work of “affect” as delivered via telehealth technologies was able to bolster their patient-centered approach to engaging rural Veterans. By digging deeper into their coaching repertoire and enlisting a variety of flexible strategies and technologies, WH coaches promoted increased self-management of rural Veterans, while providing social support.

The increased use of remote technologies that resulted from COVID-19 [54] has the potential to extend the meaning of patient-centered healthcare by enhancing the providers’ ability to see a patient within their home environment, and thus, potentially increasing their understanding of patients’ needs specific to their unique environmental context [54]. Ensuring that front-line health professionals have access to resources for addressing wide-ranging patient needs, including SDOHs, is key in a post-pandemic context [63]. Moreover, in a post-COVID-19 world, as more healthcare is delivered with the facilitation of remote technology, it is imperative that people who are rural and have constrained options for engaging through remote technologies, are not left behind.

Finally, we found that the COVID-19 pandemic exacerbated existing concerns for Veteran mental health. Veteran mental health is longstanding primary concern within the VHA [56]. Research efforts around the utilization of mental health services by rural Veterans [57], for conditions such as depression, anxiety, and PTSD, are ongoing, as are the development of clinical programs [58,59,60,61]. This concern underpinned our WH coaches’ extraordinary efforts in increasing their social support of the Veteran patients, which was intended to bolster Veterans’ ability to cope with stressors brought on by pandemic. This finding is consistent with published models indicating how social support buffers stress effects on wellbeing [47,64], and provides an in-depth illustration of coaches’ use of social supports to attempt to bridge the impact of COVID-19 on Veterans’ health.

### Limitations and Future Work

Findings provide the first appraisal of the VHA WH initiative from the perspective of coaches working specifically with rural Veterans during the COVID-19 pandemic. However, viewpoints were not obtained from Veterans or non-coaches regarding the impact of the pandemic on their WH experience which limits our ability to fully describe. Findings also contribute in-depth insight into a wide range of processes used by practicing VHA coaches; they do not, however, provide insights into practice trends that may have transferability beyond our sample. Future research studies are warranted, including those encompassing Veterans’ and other allied health professionals’ perspectives.

## 5. Conclusions

This paper explored perspectives of Whole Health coaches on meeting the needs of rural Veterans during the COVID-19 pandemic. Coaches perceived that key social determinants of health were negatively impacted for many rural-dwelling Veterans during the pandemic in the areas of social and community context, health and health care, and neighborhood and built environment. In their ingenuity and commitment to supporting rural Veterans, coaches expanded their forms of support to respond to the wide-ranging needs of rural Veterans and attempted to bridge risk factors that were exacerbated for Veterans during the pandemic. Our findings suggest that health coaches may be well positioned to address Veterans’ health within a broader definition of wellness by leveraging additional forms of social support and tapping into expanded networks during times of crisis; however, more research is needed to examine the role and scope of coaches during such pivotal times.

## Data Availability

The datasets presented in this article are not readily available because supporting data cannot be made openly available due to VHA privacy requirements. Requests to access the datasets should be directed to jennifer.hale-gallardo@va.gov.
